# IL-17C/IL-17RE: Emergence of a Unique Axis in T_H_17 Biology

**DOI:** 10.3389/fimmu.2020.00341

**Published:** 2020-02-26

**Authors:** Jasper F. Nies, Ulf Panzer

**Affiliations:** ^1^Translational Immunology, III. Department of Medicine, University Medical Center Hamburg-Eppendorf Hamburg, Hamburg, Germany; ^2^Hamburg Center of Translational Immunology (HCTI), University Medical Center Hamburg-Eppendorf, Hamburg, Germany

**Keywords:** IL-17C, IL-17RE, immunity, inflammation, Th17

## Abstract

Therapeutic targeting of IL-17A and its receptor IL-17RA with antibodies has turned out to be a tremendous success in the treatment of several autoimmune conditions. As the IL-17 cytokine family consists of six members (IL-17A to F), it is intriguing to elucidate the biological function of these five other molecules to identify more potential targets. In the past decade, IL-17C has emerged as quite a unique member of this pro-inflammatory cytokine group. In contrast to the well-described IL-17A and IL-17F, IL-17C is upregulated at very early timepoints of several disease settings. Also, the cellular source of the homodimeric cytokine differs from the other members of the family: Epithelial rather than hematopoietic cells were identified as the producers of IL-17C, while its receptor IL-17RE is expressed on T_H_17 cells as well as the epithelial cells themselves. Numerous investigations led to the current understanding that IL-17C (a) maintains an autocrine loop in the epithelium reinforcing innate immune barriers and (b) stimulates highly inflammatory T_H_17 cells. Functionally, the IL-17C/RE axis has been described to be involved in the pathogenesis of several diseases ranging from infectious and autoimmune conditions to cancer development and progression. This body of evidence has paved the way for the first clinical trials attempting to neutralize IL-17C in patients. Here, we review the latest knowledge about identification, regulation, and function of the IL-17C/IL-17receptor E pathway in inflammation and immunity, with a focus on the mechanisms underlying tissue injury. We also discuss the rationale for the translation of these findings into new therapeutic approaches in patients with immune-mediated disease.

## Introduction

The discovery of T_H_17 cells as a novel subset of CD4^+^ T cells in 2005 (1) lead to a paradigm shift in the field of immunology. Our previously incomplete and inconsistent understanding of many diseases' pathogenesis was manifold enhanced thanks to rigorous examination of this new T cell lineage. These discoveries are not only important for basic immunological research, but drugs targeting T_H_17-related molecules have had a significant impact on the treatment of immunological diseases (2–4).

**Figure 1:**
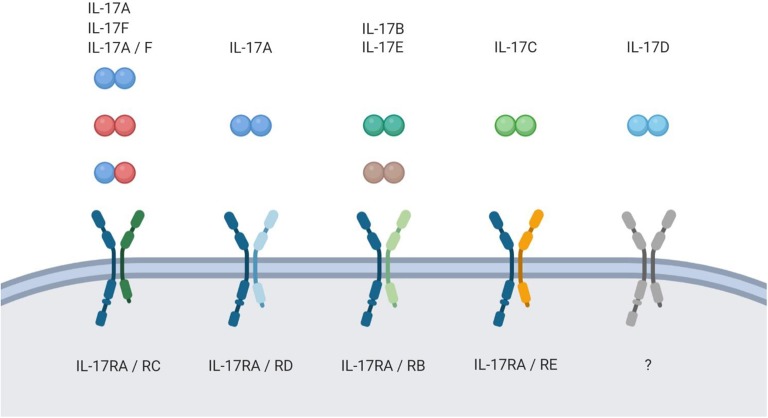
The IL-17 family. Schematic overview of the IL-17 family members and their respective receptor complexes.

As the name of the T_H_17 cells was coined by their characteristic production of the highly inflammatory cytokine IL-17A upon activation, most scientific effort has been put into understanding the biological activity of this protein. However, five more cytokines with structural similarity to IL-17A have been identified (IL-17B-F). In this six-member cytokine family, IL-17A is best characterized, followed by the very closely related IL-17F. Structurally, all members of the IL-17 cytokine family are homodimers in their biologically active form, yet one heterodimer consisting of IL-17A and IL-17F (IL-17A/F) is described (5, 6). The proteins bind to heterodimeric receptor complexes to induce signaling in their target cells. Most of those complexes consist of the ubiquitously expressed subunit IL-17RA and a second, ligand-specific subunit (IL-17RB-RE) (7–11). IL-17D remains an orphan ligand in the cytokine family (Figure 1).

In line with the current understanding of T_H_17 cells being a highly inflammatory lineage, IL-17A and F induce several inflammatory pathways. Most markedly, their binding to the receptor complex IL-17RA/RC, which is predominantly expressed on epithelial cells, leads to upregulation of cytokines, anti-bacterial peptides, and chemokines. The chemokines then recruit innate immune cells like neutrophils which potently enhance the inflammatory reaction. Thus, it is fair to say that by now we have got a good grasp of how IL-17A and F unfold their inflammatory effect.

The role of the remaining four IL-17 family members has long been considered rather elusive. However, the last years have shed a little more light on the IL-17C/RE axis, which unveiled some unique features.

In this review, we provide an overview of expression patterns and the functional importance of IL-17C and its receptor IL-17RE in immunological diseases, present a hypothesis of how the IL-17C/RE axis mediates its inflammatory effect, summarize intracellular signaling pathways, and give an outlook on translational approaches.

## Identification of the IL-17C/RE Axis

### First Characterization of IL-17C

In 2000, the cytokine IL-17C has first been identified by a homology-search for proteins similar to IL-17A (12). *IL17C* is located on chromosome 16q24, is 1.1 kb long, and the protein IL-17C shares roughly 27% amino acid identity with IL-17A. Interestingly, after stimulation no induction of *IL17C* mRNA was observed in CD4^+^ cells, which are the main source of IL-17A and F. This was the first evidence that IL-17C seems to assume a unique role in the IL-17 family. In an initial functional analysis of the protein, the authors showed that IL-17C stimulated the monocytic cell line THP-1 to release TNF-α and IL-1β.

### IL-17C Is Expressed by Epithelial Cells and Not by Hematopoietic Cells

Unlike what is known about the other IL-17 family members, many studies suggest that *IL17C* is not expressed by leukocytes, but by non-hematopoietic cells.

The characteristic production of IL-17A by a subset of CD4^+^ cells has led to the name of T_H_17 cells, which emerged to be a distinct lineage apart from the classical dichotomy of T_H_1 and T_H_2 cells. However, not only CD4^+^ T cells produce IL-17A, but also CD8^+^ T cells (13), γδ T cells (14, 15), invariant natural killer T cells (iNKT) (16), group 3 innate lymphoid cells (ILCs) (17), and even B cells (18).

In contrast, *IL17C* is expressed by epithelial cells. In a model for psoriasis, keratinocytes are the main source of IL-17C (19). Several groups confirmed this *IL17C* expression in keratinocytes (20–23). Other epithelial cells producing the cytokine include colonic epithelial cells (9), resident kidney cells (24, 25), and respiratory epithelial cells (26–28).

Although this strong evidence points to epithelial cells as the main source of IL17C, its expression has also been found in leukocytes (29, 30) and smooth muscle cells (31).

#### IL-17C and the Microbiome

T_H_17 biology is closely linked to the microbiome as it influences T_H_17 cell development: Experiments with antibiotic treatment or germ-free mice drastically reduced intestinal T_H_17 cells (32, 33). However, specific bacteria are required for proper induction of this cell type. Segmented filamentous bacteria (SFB) can potently induce the T_H_17 cell development (34, 35), while *Bacteroides fragilis* suppresses this differentiation (36). Thus, changes in the gut flora influence the development of T_H_17 cells, which can both aggravate or ameliorate extra-intestinal T_H_17-driven autoimmunity (37).

Even though T_H_17 cells themselves are not the source of IL-17C, intestinal bacteria still seem to play a role for *IL17C* expression in the gut. Antibiotic treatment of mice blocked the induction of *Il17c*. IL-23 and IL-22 were reported to be dispensable, but TLR-MyD88 signaling in gut resident cells was essential for the induction of *Il17c* expression. In the same experiment, the authors identified MyD88 as being essential for proper induction of *Il17a*, but not *Il17c*, in hematopoietic cells (38). Co-culture of murine colonic epithelial cells with *Citrobacter rodentium* induces IL-17C production in those cells. Specifically, Lipopolysaccharide (LPS) and flagellin are two pattern-associated molecular patterns (PAMPs) that can be recognized by toll-like receptors (TLRs) and culturing the cells with those components alone resulted in strong IL-17C production (9). A change in *Il17c* expression was not observed in any of the analyzed leukocyte populations (T lymphocytes, B lymphocytes, intraepithelial lymphocytes, lamina propria mononuclear cells). This finding was validated by the fact that no difference in *Il17c* induction was seen between wildtype and recombination-activating gene 1 (*Rag1*) deficient mice. Within the non-leukocytic cell populations in the colon, *Il17c* induction was indeed limited to only the colonic epithelial cells since no mRNA upregulation *Il17c* was seen in colonic stromal cells after infection (9). Those findings indicate that TLR activation by microbiota in the gut is important for both IL-17A and IL-17C, albeit the source of those cytokines is located to different cell types: Hematopoietic cells and gut resident epithelial cells, respectively.

Thus, epithelial cells are the main source of the cytokine in different tissues. That stands in stark contrast to the cellular source of other cytokines of the IL-17 family, which are mainly expressed by leukocytes.

### *IL17C* Is Upregulated Early in Disease

Regarding the temporal expression of *IL17C*, current data point to an early upregulation during disease. *Il17c* mRNA was strongly upregulated after 4 days in the colons of bacterially infected mice, while *Il17a* expression peaked at day 12 (9). Ramirez-Carrozzi et al. analyzed the kinetics of *Il17c* expression in detail: *in vitro* stimulation of HCT-15 cells with heat-killed *E. coli* lead to a rapid expression of the cytokine after 1 h and murine skin challenged with imiquimod showed strong *Il17c* expression after 2 days. In the DSS-colitis model, the authors found induction of *Il17c* expression after 2 days in colons and mesenteric lymph nodes, but upregulation of *I17a* and *Il17f* mRNA transcripts was not detected before day 6 (19). In the nephrotoxic nephritis (NTN) mouse model for crescentic glomerulonephritis, we showed that *Il17c* is upregulated as early as 12 h after induction of the disease, while *Il17a* and *Il17f* expression starts after a couple of days (24).

### IL-17C Binds to the Receptor Complex IL-17RA/RE

The group that first described IL-17C also suggested that IL-17C does not bind to IL-17RA, but to another receptor. Expressing a His-tagged and metabolically labeled form of the extracellular domain of IL-17RA in 293T cells, precipitation with IL-17A was observed as expected, but no precipitation could be detected during incubation with IL-17C (12). Six years later, another group discovered this receptor, which has been named IL-17RE. Murine IL-17RE shares 40% DNA and 18% amino acid sequence with IL-17RC (39). However, the authors did not yet identify a ligand binding to this receptor subunit. The receptor was found to be expressed in lung, kidney, stomach, intestine, and testis of mice and to have six different isoforms.

Several groups described that IL-17C is the specific ligand for IL-17RE in 2011: Transfection of 293T cells with the receptor subunits IL-17RA-RE revealed that IL-17C seems to bind exclusively to IL-17RE (40). Another group used a similar approach by analyzing the binding of Flag-tagged human IL-17C to HEK293 cells overexpressing each of the five IL-17 receptor subunits. In contrast to the findings of the first characterization of IL-17C (no binding to IL-17RA) (12), flow cytometer examination of the cells after incubation with IL-17C showed binding to both IL-17RA and IL-17RE, but none of the other receptor subunits. Also, no interactions were found between IL-17RE and any of the other IL-17 cytokine family members (19).

Song and colleagues used glutathione S-transferase precipitation to demonstrate that IL-17C associates not only with IL-17RE but with a heterodimeric receptor complex consisting of IL-17RA and IL-17RE (9). Using a blocking antibody against IL-17RA during stimulation of keratinocytes with IL-17C, a dose-dependent inhibition of IL-17C-induced G-CSF and β-defensin-2 expression was observed, underlining the functional dependence on IL-17RA (19).

### The IL-17RA/RE Receptor Complex Is Expressed on Both Epithelial and T_H_17 Cells

Interestingly, epithelial cells—the main source of IL-17C—express the specific receptor for the cytokine. Strong *Il17re* expression has been detected in keratinocytes and colon epithelial cells (19). Reynolds et al. report expression of the receptor subunit in the colonic epithelial cell line YAMC (41). *IL17RE* is also expressed in nerve fibers of human skin after HSV-2 reactivation (21).

Apart from epithelial cells, Chang et al. *first* described that *Il17re* is expressed on T_H_17 cells. While numerous tissues express an isoform of IL-17RE that lacks the transmembrane domain, T_H_17 cells expressed high amounts of full-length *IL17RE*. This expression is strongly enhanced when the cells are stimulated with a cytokine cocktail of IL-6, TGF-β, IL-1, and IL-23 and IL-17C also induced expression of the receptor on T_H_17 cells (40). Validating this finding, our group found strong *Il17re* expression IL-17A^+^ YFP^+^ cells from IL-17A YFP^+^ fate reporter mice and in T_H_17 polarized cells (compared to T_H_0, T_H_1, and Treg cells) (24).

Similar to *IL17C*, we reported that *Il17re* was upregulated 24 h after induction of NTN (24).

In summary, epithelial cells produce IL-17C at early timepoints in disease. The cytokine signals through the heterodimeric receptor complex IL-17RA/RE. This complex is expressed by several epithelial cells themselves. Secondly, T_H_17 cells express *IL17RE* which indicates that this T cell lineage is also responsive to IL-17C (Table 1).

**Table 1:** Sources of IL-17C and IL-17RE.

**Protein**	**Cell type**	**References**
IL-17C	Keratinocytes	(19–23)
	Resident kidney cells	(24, 25)
	Colonic epithelial cells	(9)
	Respiratory epithelial cells	(26–28)
	Smooth muscle cells	(31)
	Leukocytes	(29, 30)
IL-17RE	T_H_17 cells	(24, 40)
	keratinocytes	(19)
	Colonic epithelial cells	(19, 41)
	Skin nerve fibers	(21)

*Overview of cell types producing IL-17C and IL-17RE*.

## Infection and Autoimmunity

Many studies report that *IL17C* expression is upregulated at an early stage in both infectious and autoimmune diseases. This suggests that it is involved in the innate first-line immunity in the pathogenesis of those conditions. Intriguingly, IL-17C also plays an important role in the initiation of the adaptive immune response later. First, we will take a closer look at the role of the IL-17C/RE immune axis in infectious diseases. Second, we will zoom in on autoimmune conditions.

### IL-17C/RE Signaling Induces Innate Immune Functions in Bacterial, Fungal, and Viral Infections

Signaling through the IL-17C/RE is involved in host defense against foreign pathogenic microorganisms. In the following paragraphs, we will summarize the role of the axis in bacterial, fungal, and viral infections.

#### Bacterial Infections

The IL-17C/RE axis plays a significant role in several bacterial infection models. Mice infected with the intestinal pathogen *Citrobacter rodentium* showed upregulation of *Il17c* mRNA in the colon (9). *Ex vivo* cultured murine colon tissue and colonic epithelial cells showed marked mRNA expression of antibacterial peptides, inflammatory cytokines, and chemokines after stimulation with IL-17C. Clinically, lack of signaling through the IL-17C/RE axis modeled with *Il17re*^−/−^ mice lead to decreased mRNA levels of said molecules and failure to clear the infection. This resulted in loss of body weight, higher intestinal and splenic weight, increased bacterial burden, and death. Interestingly, there was no difference when the cells were treated with IL-17A or F, which indicates that IL-17RE is dispensable for these two cytokines.

In a model of acute colitis, *I17c*^−/−^ mice challenged with dextran sulfate sodium (DSS) had a significantly worse outcome than mice with physiological IL-17C production, which is reflected by earlier and more pronounced weight loss and colonic shortening. The authors explain this observation with the fact that IL-17C induced mRNA expression of tight-junction molecules, which are essential for the integrity of the colonic mucosal barrier (41).

Another group examined the role of IL-17RE in this model confirming those findings of the IL-17C/RE axis assuming a crucial role in protection against bacteria-driven DSS-induced colitis (19).

The immune axis also plays a role in the defense against airway infections with *Pseudomonas aeruginosa* and *Haemophilus influenza* (26, 27).

#### Fungal Infections

The impact of IL-17C has also been examined in fungal infections. Huang and colleagues reported that IL-17C is required for a lethal course of systemic infection with *Candida albicans* in mice since *II17c*^−/−^ mice displayed increased survival and less severe functional and morphological kidney damage (25). This is in contrast to the function of IL-17A in this model: While *Il17a* overexpression protects the mice, lack of signaling through IL-17RA results in increased susceptibility to this fungal infection (42). Similarly, patients with Job's syndrome, a condition with T_H_17 cell defects, are also at great risk to suffer from such fungal infections (43, 44). Another study reported that IL-17C is not involved in immunity to systemic, oral and dermal candidiasis (45). Even though *Il17c* mRNA expression was upregulated 2 days after exposure to the fungus, the group did not observe a difference in clearance of the infection or gene expression profiles between mice lacking IL-17C or IL-17RE compared to a wildtype control group.

#### Viral Infections

Two studies investigated the role of IL-17C/RE in viral infections. Peng et al. showed that IL-17C was the only IL-17 family cytokine that was induced in keratinocytes from human genital skin biopsies during recurrent HSV-2 reactivation. Also, cultured human keratinocytes produced IL-17C in response to infection with HSV-2. Since cutaneous nerve fibers expressed *IL17RE* and *ex vivo* application of IL-17C reduced apoptosis in the nerve cells, the authors hypothesize that keratinocyte-derived IL-17C serves as a protective agent for nerve fibers during HSV-2 reactivation in the skin (21). Another group recently analyzed the effects of IL-17C in *in vitro* virus-bacteria coinfection of human bronchial epithelial cells to assess the cytokine's role in COPD exacerbations. A challenge with both pathogens resulted in a synergistic induction of IL-17C. Interestingly, tissue from healthy smokers released little IL-17C upon exposure to the pathogens, but epithelial cells from COPD patients released significantly more. Thus, the IL-17C/RE axis might be involved in the pathogenesis of COPD exacerbations of mixed upper airway infections (46).

### Several T_H_17-Driven Autoimmune Diseases Are Exacerbated by IL-17C/RE

Inflammation orchestrated by T_H_17 cells is a hallmark of various autoimmune conditions like rheumatoid arthritis, psoriasis, multiple sclerosis, autoimmune kidney diseases, and autoimmune hepatitis.

In 2007, Yamaguchi et al. attributed IL-17C a role in the pathogenesis of collagen-induced arthritis (30). Mice adoptively transferred with CD4^+^ T cells, which were retrovirally transduced with either IL-17A, B, C, or F, had significantly higher arthritis scores than those that got cells transduced with an empty vector.

Several studies also evaluated the role of IL-17C in skin inflammation complementing the picture of IL-17C-induced auto-aggression. Johansen et al. first showed that *IL17C* mRNA and protein levels were increased in the skin of patients with psoriatic lesions compared to non-lesional skin (47). In fact, IL-17C is by far the most abundant IL-17 cytokine found in the skin of such skin lesions: Its protein levels were reported to be roughly 125-fold higher than those of IL-17A in the lesions (48). Transgenic mice lacking *Il17c, Il17ra*, or *Il17re* display a less severe course of imiquimod-induced psoriasis (19, 49) while an overexpression of *Il17c* in skin keratinocytes lead to spontaneous development of psoriasiform skin lesions (48). IL-17C also drives inflammation in atopic dermatitis as *IL17C* expression was increased in lesional skin of patients and blocking IL-17C with an antibody ameliorated skin inflammation in one mouse model for psoriasis and two models for atopic dermatitis (50).

Also, IL-17C/RE signaling aggravates the course of experimental autoimmune encephalitis (EAE) (40). *Il17c*^−/−^ mice were less prone to develop the disease and those that did showed less pronounced clinical manifestations of the inflammation. Vice versa, increased signaling through the axis in transgenic mice overexpressing *Il17re* in CD4^+^ cells lead to a worse clinical situation of the animals.

We have recently described that the serum levels of IL-17C are significantly higher in patients with ANCA-associated glomerulonephritis compared to a healthy control group, which was not true for IL-17A, F, and B. We showed the pro-inflammatory role of IL-17C in established mouse models for lupus nephritis and crescentic glomerulonephritis. In accordance with the mentioned previous studies, our experiments showed expression of *Il17re* by T_H_17 cells and significantly less T_H_17 cells in inflamed kidneys of both *Il17c*^−/−^ and *Il17re*^−/−^ mice (24).

Two studies investigated the involvement of the IL-17C/RE axis in autoimmune hepatitis. One group found evidence that IL-17C stimulates intrahepatic CD4^+^ T cells to release IL-2 with subsequent NK-cell mediated liver damage. In this study, lesser levels of GOT and GPT in sera of *Il17c*^−/−^ and *Il17re*^−/−^ mice were found compared to wildtype mice (51). However, another group found no differences in GOT and GPT activities and granulocyte infiltration into the liver between *Il17c*^−/−^ and wildtype mice in the same model (52).

Further diseases involving the IL-17C/RE axis include psoriasiform skin lesions in inflammatory bowel disease (IBD) patients under anti-TNF-α treatment (53), recurrent aphthous ulcers (20), LPS-induced endotoxin shock (52), and different forms of cancer (38, 54–56) (Tables 2, 3).

**Table 2:** Main findings of experimental data on IL-17C and IL-17RE.

**Disease model**		**Mice used**	**Main phenotype of investigated group**	**References**
Experimental autoimmune encephalitis (EAE)		*Il17c^−/−^*	Less clinical manifestation, lower mortality	(40)
		*Il17re* overexpressing CD4^+^ T cells adoptively transferred to wildtype C57Bl/6	Increase in EAE symptoms	
Nephrotoxic Nephritis (NTN)		*Il17c^−/−^* *Il17re^−/−^*	Reduced functional and morphological kidney damage, less renal Th17 infiltration	(24)
Pristane-induced lupus nephritis		*Il17c^−/−^*	Reduced functional and morphological kidney damage	(24)
Psoriasis	Imiquimod-induced	*Il17c^−/−^* *Il17re^−/−^*	Less severe course of the disease	(19, 49)
	Il-17c- induced	*Il17c* overexpression in keratinocytes of wildtype C57Bl/6	Spontaneous development of psoriasiform skin lesions	(48)
	IL-23-induced	BALB/c	Reduced ear swelling and acanthosis under anti-IL-17C treatment	(50)
Con-A-induced autoimmune hepatitis		*Il17c^−/−^* *Il17re^−/−^*	Lesser levels of GOT and GPT, attributed to inhibited NK-cell mediated liver damage	(51)
		*Il17c^−/−^*	No difference in GOT and GPT levels or hepatic granulocyte infiltration	(52)
Collagen-induced Arthritis (CIA)		*Il17c* overexpressing CD4^+^ T cells adoptively transferred to DBA1 mice *Il17c* BM chimeric mice	Higher arthritis scores than control	(30)
Atopic dermatitis	MC903-induced	BALB/c	Less severe ear swelling under anti-IL-17C treatment	(50)
	Flaky tail	Flaky tail (Matt^ma^/^ma^Flg^ft/ft^)	Less hair loss and excoriation and ameliorated blepharitis under anti-IL-17C treatment	
Dextrane sulfate sodium (DSS) induced colitis		*Il17c^−/−^* *Il17re^−/−^*	More pronounced body weight loss and colonic shortening due to lesser expression of antibacterial, inflammatory, and tight-junction molecules	(19, 41, 52)
*Citrobacter rodentium* infection		*Il17re^−/−^*	More body weight loss, higher intestinal and splenic weight, higher bacterial burden, higher mortality	(9)
*Pseudomonas aeruginosa* airway infection		*Il17c^−/−^*	Increased survival	(26)
Systemic *Candida albicans* infection		*Il17c^−/−^*	Increased survival and less severe kidney damage	(25)
Systemic, oral and dermal Candidiasis		*Il17c^−/−^* *Il17re^−/−^*	No difference between knockout and wildtype groups	(45)
LPS-induced endotoxin shock		*Il17c^−/−^*	Higher resistance to endotoxin-induced shock.	(52)

*Summary of current data on the IL-17C/RE axis in mouse models*.

**Table 3:** IL-17C/RE data on human samples.

**Disease**	**Main finding**	**References**
Psoriasis	Elevated levels of *Il17c* mRNA and IL-17C protein in lesional patient skin. Impaired *Il17re* expression in those lesions.	(47)
	IL-17C most abundant IL-17 cytokine in lesional skin (125-fold of IL-17A)	(48)
ANCA-associated glomerulonephritis	IL-17C as the only IL-17 cytokine with elevated serum protein levels	(24)
Atopic dermatitis	Increased *Il17c* expression and positive immunohistochemistry staining for IL-17C in skin of atopic dermatitis patients	(50)
Recurrent aphthous ulcers (RAU)	Human oral keratinocytes stained positive for IL-17C in RAU lesions of patients and expressed TNF-α in response to IL-17C *in vitro*	(20)
Anti-TNF-α-induced psoriasiform skin lesions in Crohn's disease	High IL-17C protein concentrations in skin lesions	(53)
HSV-2 reactivation in genital skin	Protective effect of IL-17C on skin neurons	(21)
*Pseudomonas aeruginosa* airway infection	Enhanced inflammatory response to infection by human epithelial cell line	(27)
Virus-bacteria coinfection in COPD	Coinfection led to synergistic upregulation of *Il17c* in human bronchial epithelial cells; stimulation with IL-17C upregulated chemokines.	(46)

*Summary of current human data on the IL-17C/RE axis*.

## Mechanisms of IL-17C/RE Driven Inflammation

Mechanistically, a body of evidence suggests that IL-17C exerts two important immunological effects: (a) In an autocrine feedback loop with epithelial cells, IL-17C strengthens innate barriers against infectious agents. (b) Boosting T_H_17 cell function, IL-17C also stimulates the adaptive immune system to efficiently fight off infections. Yet, those pathways harbor the risk of T_H_17-driven autoimmunity.

As IL-17A has been studied much more extensively as IL-17C and acts on epithelial cells, it is worthwhile to recapitulate the signaling of IL-17A through IL-17RA.

The similar expression of fibroblast growth factor and IL-17R (SEFIR) domain is highly conserved within the IL-17 receptor family and structurally similar to the Toll/IL-1R (TIR) domain found in TLRs and the IL-1β receptor (57). Yet, IL-17 signaling employs an adaptor protein unique to IL-17 signaling called ACT1, which also carries the SEFIR domain. The adaptor protein can then bind several intracellular signaling proteins to induce several conserved signaling pathways. Pathways activated by IL-17 receptor signaling include nuclear factor kappa-light-chain-enhancer of activated B cells (NF-κB) (58), inhibitor of NF-κB ζ (IκBζ) (59), mitogen-activated protein kinase (MAPK) (60–62), and CCAAT/enhancer-binding protein (C/EBP) (63, 64). Together, these pathways mediate mitogenic signals and induce expression of pro-inflammatory cytokines and chemokines.

Another domain called TIR-like loop (TILL) domain is crucial for IL-17 signaling but is only present in the IL-17RA subunit (65). However, most of the other subunits of this family heterodimerize with IL-17RA to form a functional complex, which suggests that IL-17RA is the domain necessary for intracellular signaling. Also, the C/EBPβ activation domain (CBAD) on IL-17RA stimulates signaling through the transcription factor C/EBPβ (65), initiating one of the few known inhibitory mechanisms of IL-17 signaling (66).

A very important aspect of IL-17 signaling is synergism: *De novo* gene expression by IL-17A in target cells does not fully account for the observed strong inflammatory effect of the cytokine. Signaling through IL-17RA stabilizes mRNA transcripts of genes expressed by other strong inflammatory stimuli like TNF-α (59, 67). Ligand binding to IL-17RA recruits the kinase IKKi to phosphorylate ACT1. TRAF2 and 5 then bind to form a complex that can inhibit cleavage of mRNA (61, 68).

Thus, the full biological activity of IL-17A becomes apparent only in concert with other factors of the inflammatory milieu. Such synergetic effects have also been described between IL-17C and three other cytokines: TNF-α (19, 24, 48), IL-22 (9, 24), and IL-1β (19). However, the underlying molecular mechanisms have not specifically been studied for IL-17C/RE signaling.

### IL-17C and the Epithelial Cell

The first site of IL-17C immunity is the epithelial cell. Group-specific innate signaling pathways like the activation of TLRs in response to PAMPs induce expression of *Il17c* (9, 19, 38). Activation of TLR is one of the first responses of the immune system after contact with pathogens, which explains the early upregulation of *IL17C* in the various infectious diseases. Intracellular MyD88 signaling induced by TLR2 and 5 agonists or IL-1β stimulated the expression of *IL17C* in mucosal epithelial cells (19). Another intracellular mechanism for *IL17C* expression in response to pathogens is activation of nucleotide-binding oligomerization domain-containing protein 2 (NOD2) by *Staphylococcus aureus* (69).

There is strong evidence for a synergistic effect between TNF-α and IL-17A as IL-17A signaling stabilizes mRNA of target genes of TNF-α. Interestingly, one target gene that is synergistically induced by IL-17A and TNF-α is *IL17C* (70). However, stimulation of murine and human epithelial cells with TNF-α or IL-17A alone is also capable of upregulating *IL17C* expression (9, 19).

These findings are underlined by the fact that *IL17C* expression is decreased in skin biopsies of psoriasis patients under anti-TNF-α therapy (22). Likewise, IL-17RA blockade with Brodalumab lead to decreased levels of *IL17C* expression in psoriatic skin (71).

In terms of signaling cascades, TNF-α signaling seems to employ the p38 mitogen activated protein kinase (22) and the NF-κB pathway to enhance *IL17C* expression. Direct evidence of this are three bindings sites for NF-κB in the *IL17C* promotor (23).

Thus, both PAMPs and pro-inflammatory cytokines can induce *IL17C* expression. TNF-α and IL-17A are able to induce *IL17C* individually and a strong synergistic effect between the two cytokines drastically boosts the expression.

Binding of IL-17C to the IL-17RA/RE complex on the epithelial IL-17C-source cells forms an autocrine loop in the epithelium. Like IL-17A, IL-17C signaling through IL-17RA/RE employs the adaptor molecule ACT1 (40). The signaling cascade then activates the MAPK pathway by phosphorylation of p38, ERK, and JNK as well as the NF-κB pathway by phosphorylation of the p65 subunit and the NF-κB inhibitor IκBα (9). Also, signaling through L-17RA/RE on epithelial cells reinforces the mechanical epithelial barrier by expressing the tight-junction proteins occludin, claudin-1, and claudin-4 (41). Host defense mechanisms in epithelial cells induced by IL-17C include the expression of hBD2, S100A7/8/9, CXCL1/2/3, CCL20, TNFAIP6, and TNIP3 (19) as well as pro-inflammatory cytokines like IL-1β, IL-17A/F, IL-22, IL-6, IL-8, VEGF, and TNF-α (48). This expression profile is a potent response to actively fight off invading pathogens.

Thus, the autocrine loop of IL-17C in the epithelium is an early protective response against pathogenic alterations in the microbiome and other epithelial tissues.

### IL-17C and the T_H_17 Cell

The second site of action of IL-17C is the T_H_17 cell. We have shown that the numbers of T_H_17 cells significantly decrease in the absence of IL-17C or IL-17RE in a murine models of autoimmune kidney diseases (24). This effect of IL-17C on T_H_17 cells might be due to increased proliferation or differentiation, inhibited apoptosis, or impeded exhaustion. Other groups have investigated these intracellular effects in more detail.

In the EAE model, T_H_17 differentiation was induced by IL-17C/RE signaling via IκBζ (40). Signaling through IL-17RE lead to increased production of IL-17A, IL-17F, and IL-22. Song et al. showed that IL-17C induces the expression of anti-apoptotic factors *BCL2* and *BCL2L1* in intestinal epithelial cells (38). This anti-apoptotic effect of the IL-17C/RE axis was also seen in nerve fibers during HSV-2 reactivation (21). As mentioned before, signaling pathways of IL-17C in epithelial cells involve NF-κB and MAPK (9), which might also be true for T_H_17 cells and would be indicative of an effect on proliferation of target cells.

Many groups have shown the pro-inflammatory role of IL-17C in disease settings that are known to be driven by a strong T_H_17 cell activity. As IL-17C induces the expression of IL-17A in T_H_17 cells (40), it may be that this effect of IL-17C is dependent on IL-17A. Indeed, blockade of IL-17A with an antibody abolished the difference in renal damage between wildtype and *Il17c*^−/−^ mice in a model for crescentic glomerulonephritis (24). Thus, this stimulatory effect of IL-17C/RE on T_H_17 cells leads to higher levels of T_H_17 signature cytokines—above all IL-17A—which accounts for the strong inflammatory effect of IL-17C. As excessive T_H_17 cell activity is linked to many autoimmune diseases, IL-17C-mediated stimulation of the T_H_17 cell represents a cause for T_H_17 autoimmunity upstream of main effector cytokines like IL-17A and F.

In terms of regulating IL-17C signaling, Monin et al. identified the endoribonuclease MCP-1 induced protein 1 (MCPIP1) as a negative regulator of both IL-17A and C signaling: In a model of imiquimod-induced skin inflammation, mice deficient in MCPIP1 showed increased inflammation and upregulation of IL-17A- and IL-17C-dependent genes, but unaltered levels of IL-17A and C. This indicates that MCPIP1 influences intracellular pathways downstream of IL-17 receptor signaling as opposed to modulation of the expression of IL-17 cytokines (72). The exact mechanism of this negative regulation on IL-17A and C signaling has not been described. However, previous studies have shown that MCPIP1 hampers TLR signaling in response to LPS by degrading mRNA of *Il6* (73) and interferes with MAPK and NF-κB signaling by deubiquitination of signaling molecules (74). Even more, MCPIP1 degrades *Il17ra* and *Il17rc* mRNA (75) and MCPIP1 deficiency boosts T_H_17 effector functions (76), which underlines its regulatory effect in IL-17 signaling.

**Figure 2:**
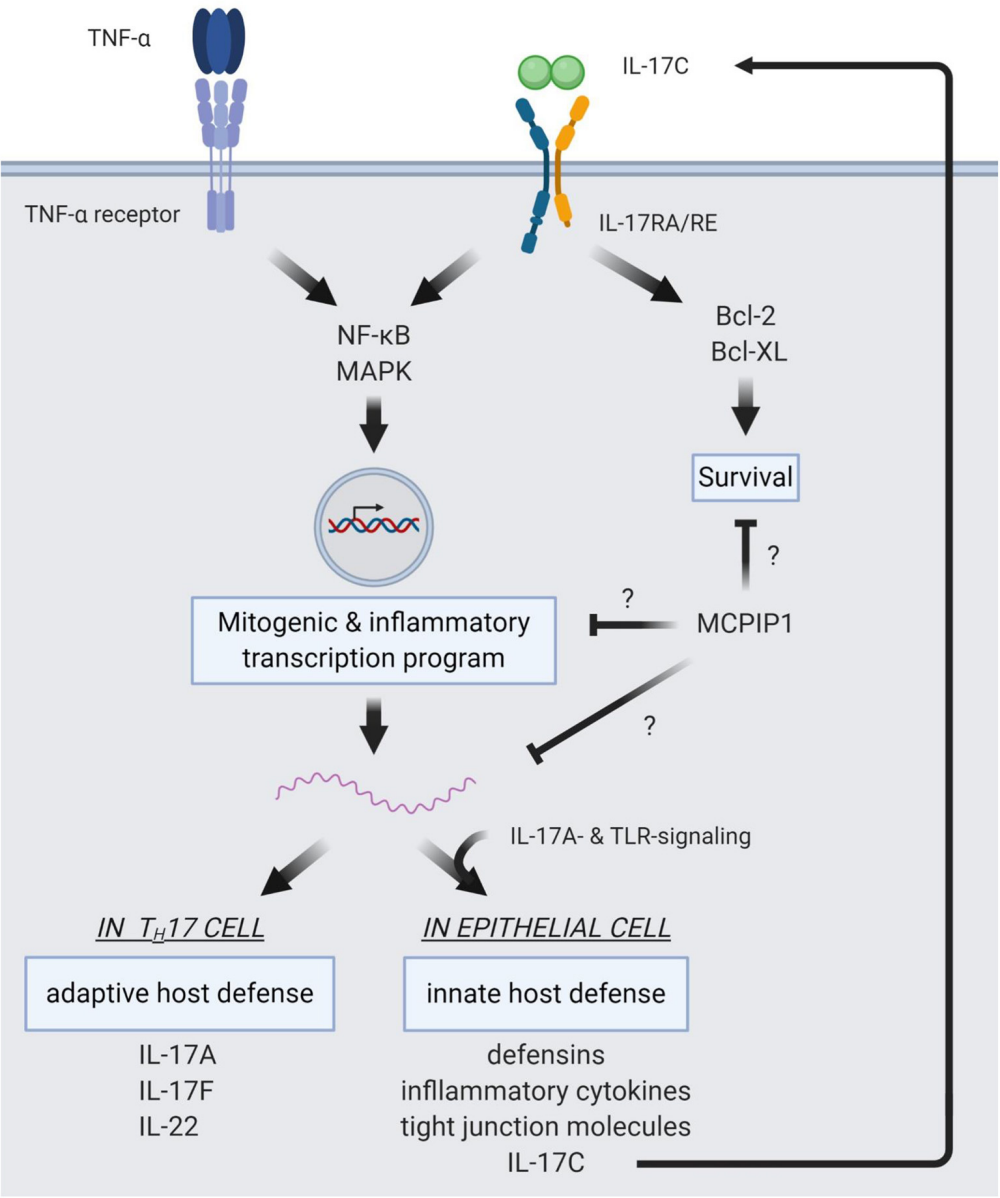
Intracellular pathways of IL-17C signaling. IL-17C signaling induces NF-κB and MAPK signaling pathways. This induction shows a synergistic effect with TNF-α signaling, resulting in strong induction of a mitogenic and pro-inflammatory expression profile. In epithelial cells, target genes of IL-17C/RE signaling are defensins, inflammatory cytokines, and tight junction molecules to reinforce innate host barriers in response to pathogens. Also, IL-17C expression is upregulated in the epithelium and subject to a synergism of IL-17A- and TLR-signaling. IL-17C is then released from the epithelial cell and binds to the IL-17RA/RE receptor complex expressed on the same cell, forming an autocrine loop. In T_H_17 cells, IL-17C induces expression of *IL17A, IL17F*, and *IL22*, boosting adaptive defense mechanisms. IL-17C also activates anti-apoptotic pathways via Bcl-2 and Bcl-X_L._ MCPIP1 is a regulator of IL-17C/RE signaling, but the distinct mechanisms of this negative regulation are not yet elucidated.

Figure 2 summarizes intracellular signaling pathways of IL-17C.

**Figure 3:**
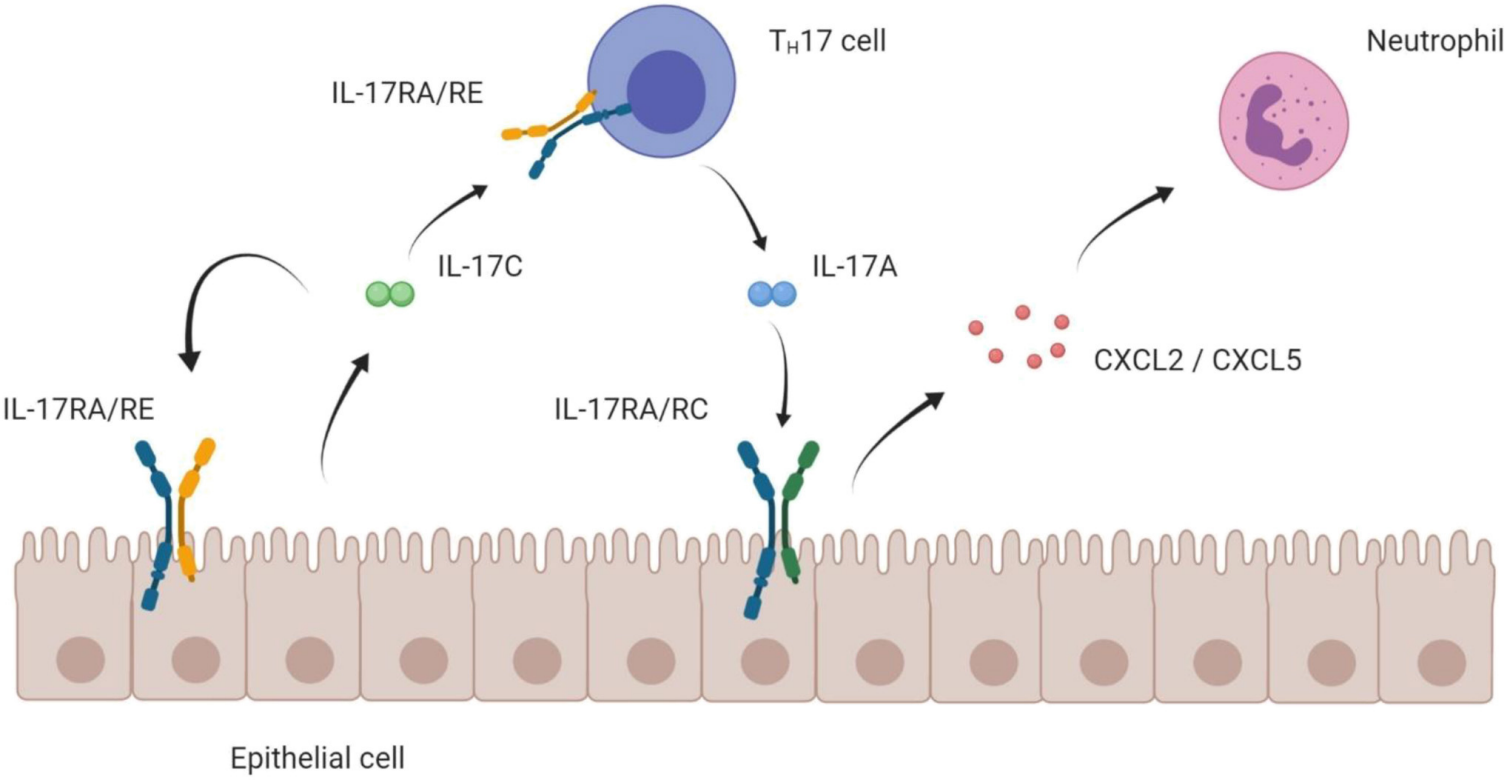
Mode of action of IL-17C on epithelial and T_H_17 cells. Schematic hypothesis of the pro-inflammatory mode of action of the IL-17C/RE axis. IL-17C is mainly expressed by epithelial cells. Expressing the IL-17RA/RE receptor complex, both the epithelial cell itself and T_H_17 cells are targets for IL-17C. Boosting expression of *IL17A* in T_H_17 cells, IL-17C indirectly enhances epithelial expression of chemokines that attract neutrophils, which ultimately cause a strong inflammatory reaction.

Taken together, IL-17C assumes a position at the interface of innate and adaptive immune system: It is upregulated during early stages of disease and reinforces innate defense lines in the epithelium via an autocrine loop. Its stimulatory action on the T_H_17 cells induces the adaptive immune response and can trigger autoimmune disease (Figure 3).

## First Steps in Therapeutic Targeting of the IL-17C/RE Axis

Antibodies targeting T_H_17 cell functions are already in clinical use for a host of autoimmune disorders like psoriasis, psoriatic arthritis, and IBD.

Ustekinumab is a monoclonal antibody directed against the p40 subunit which is shared by the cytokines IL-12 and IL-23. It has been shown to be very successful in the treatment of psoriasis and psoriatic arthritis (77, 78) and is approved for Crohn's disease (79).

Other antibodies directly targeting IL-17A (Secukinumab, Ixekizumab) or the receptor IL-17RA (Brodalumab) show astonishing effects in psoriasis patients (4, 80, 81). Other indications are psoriatic arthritis (2, 3, 82) and ankylosing spondylitis (83).

Neutralizing IL-17C is an intriguing approach in the treatment of autoimmune diseases as it might hamper T_H_17 function in general and not only the impact signaling of the signature cytokine IL-17A. Indeed, the first clinical studies with an anti-IL-17C-neutralizing antibody have been started in patients with atopic dermatitis (84) after trials in murine models showed promising results (50).

Interestingly, targeting the cytokine IL-17A or its receptor IL-17RA aggravates symptoms in IBD patients (85, 86). This shows that intervening in those signaling pathways might not be as straightforward as initially thought. Thus, it might be possible that the protective role that IL-17C plays for the integrity of epithelial barrier function exceeds its pathological effect for T_H_17 stimulation in autoimmunity. Disrupting the autocrine loop of the epithelial cells with an antibody might lead to unwanted adverse effects like gastrointestinal or respiratory infections. Inhibiting the T_H_17 cell function obviously harbors the risk of a general susceptibility to infections with extracellular bacteria and fungi.

## Discussion

In summary, IL-17C is a homodimeric cytokine that is expressed by non-hematopoietic—mainly epithelial—cells. It binds to its heterodimeric receptor complex IL-17RA/RE that is expressed on both a variety of epithelial cells and T_H_17 cells. Compared to other IL-17 cytokine family members, *IL17C* is upregulated at early stages of the diseases and plays two roles. (a) In an autocrine manner it sustains barrier integrity of epithelial cell layers and thus supports the innate immune system to keep infections in check. (b) By binding to IL-17RE on T_H_17 cells, IL-17C also stimulates the adaptive immune response to potently fight off invading pathogens. The downside of this mode of action is the risk of immunological derailment, leading to autoimmune conditions.

Intracellular signaling of IL-17C/RE involves anti-apoptotic Bcl-2 and Bcl-X_L_ as well as the NF-κB and MAPK pathways to promote proliferation and host defense. The induction of *IL17C* has been shown to be dependent on TLR signaling and pro-inflammatory cytokines. *IL17C* expression is subject to a synergism between TNF-α and IL-17A, presumably due to mRNA stabilization by IL-17A. To date, the molecular mechanisms of described synergisms between IL-17C and other cytokines (TNF-α, IL-22, and IL-1β) have not specifically been investigated.

Being an inflammatory mediator upstream of T_H_17 effector cytokines, IL-17C represents an interesting target for pharmacological intervention. The first clinical trials have been started for atopic dermatitis and data from human samples suggest transferability of experimental data to the clinical setting for some diseases (24, 48, 53).

The first translational approaches to pharmacologically exploit the IL-17C/RE axis are on the way. We believe that the main potential of such interventions lies in the treatment of autoimmune disorders. Yet, a lot of experimental data on more disease settings requires further analyses of human samples to investigate potential patient populations for this kind of treatment.

## Author Contributions

UP and JN wrote the manuscript and designed the figures.

### Conflict of Interest

The authors declare that the research was conducted in the absence of any commercial or financial relationships that could be construed as a potential conflict of interest.
